# Outstanding Reviewers for *RSC Chemical Biology* in 2022

**DOI:** 10.1039/d3cb90021e

**Published:** 2023-06-26

## Abstract

We would like to take this opportunity to highlight the Outstanding Reviewers for *RSC Chemical Biology* in 2022, as selected by the editorial team for their significant contribution to the journal.
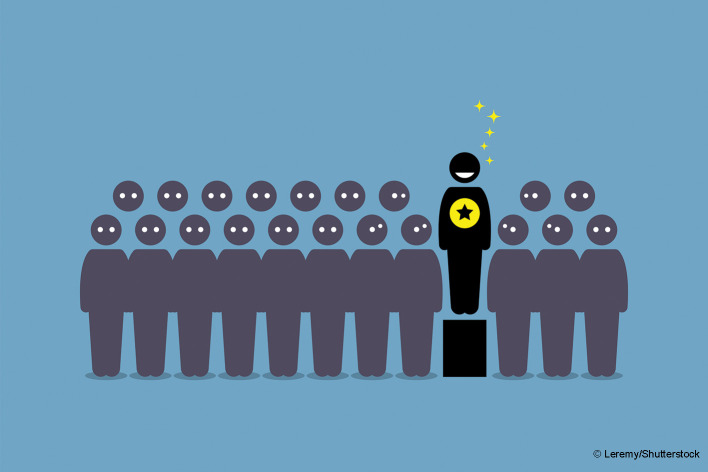

We would like to take this opportunity to thank all of *RSC Chemical Biology*'s reviewers for helping to preserve quality and integrity in chemical science literature.

We would also like to highlight the Outstanding Reviewers for *RSC Chemical Biology* in 2022. Each one of our outstanding peer reviewers has been carefully selected by our editorial team and includes active researchers who have made significant contributions to peer review by providing exceptionally thorough and detailed reports and making additional efforts to aid authors in improving their manuscripts.

 

Dr Anne C. Conibear

Vienna University of Technology

ORCID: 0000-0002-5482-6225

 

Dr Stephan Hacker

Leiden University

ORCID: 0000-0001-5420-4824

 

Dr Markus Kaiser

Universität Duisburg-Essen

ORCID: 0000-0002-6540-8520

 

Dr Jared Lewis

Indiana University

ORCID: 0000-0003-2800-8330

 

Dr Daisuke Miyoshi

Konan University

ORCID: 0000-0002-4308-0499

 

Dr Christian Adam Olsen

University of Copenhagen

ORCID: 0000-0002-2953-8942

 

Dr Steven H. L. Verhelst

Katholieke Universiteit Leuven

ORCID: 0000-0002-7400-1319

 

We would also like to thank the *RSC Chemical Biology* Editorial Board and Advisory Board and the chemical biology community for their continued support of the journal, as authors, reviewers and readers.

We continue to work on improving the diversity of our reviewer pool to reflect the diversity of the communities that we serve.

 

Dr Anna Rulka, Executive Editor

Dr Viktoria Titmus, Editorial Production Manager

## Supplementary Material

